# Book Review

**Published:** 1939

**Authors:** 


### Reviews of Books

**A Synopsis of Medicine.** By H. L. Tidy, M.D., F.R.C.P. Seventh Edition. Pp. xx., 1,187. Bristol: John Wright & Sons Ltd. 1939. Price 21s.—Tidy's *Synopsis of Medicine* needs no introduction. The Seventh Edition contains many new or rewritten sections. The result is a thoroughly up-to-date conspectus of internal medicine.

**Shock and Related Capillary Phenomena.** By V. H. Moon, M.D., M.Sc. Pp. xviii., 442. Illustrated. New York: Oxford University Press. 1938. Price 21s.—The spate of literature and research dealing with surgical shock during the Great War was followed by a low level in interest in the years following. We were tired of the subject. It awakened unhappy memories. Now after twenty years this book has been published, taking it up again, principally from the American point of view. Evidence is advanced that in surgical shock there is a concentration of the blood, shown alike by the hæmocrit readings, the rise in the specific gravity, and the increased hæmoglobin and red cell counts. The last is the easiest to read; the count may rise from five to nine million red cells. Fall of blood pressure is not a sign of shock, but of failure of compensation to shock; it is a late phenomenon. There is a reduced volume flow of blood before the pressure falls; the veins may be empty and contracted; the skin pale and flaccid. Shock is defined, therefore, as " a circulatory deficiency neither cardiac nor vasomotor in origin, characterized by decreased blood volume, decreased cardiac output, and by increased concentration of the blood." The reason why the circulating blood-volume is reduced is because of the opening up of all th capillaries of the body, especially of the viscera. The capillary dilatation is attributed to the chemical effect of damaged tissue cells; normally, a local damage to tissues liberates substances which dilate the local capillaries; in shock, this physiological process is exaggerated to catastrophic proportions. Blood volume is lost then, partly by pooling of the blood in the capillary backwaters, and partly by exudation of plasma into the tissues (œdema). At first general vasoconstriction of arteries and veins suffices to maintain the blood pressure later this compensation fails. The same concentration of the blood may be seen in patients with acute abdominal emergencies; for instance, in a case of acute pancreatitis, dying of shock, the red cell count was 6,400,000, and the autopsy showed congestion and œdema of the lungs with capillary hæmorrhages. The deductions drawn for prevention follow the ordinary lines: avoidance of rough surgery, warmth, saline infusion, early removal of hopelessly damaged tissues, enough morphine to relieve pain. For treatment of developed shock, vasoconstrictors are useless. Gum-saline or blood transfusion help up to a point; an oxygen tent would seem to be logical to combat the anoxhæmia, and perhaps the administration of adrenal cortex hormone.

**Ker's Manual of Fevers.** By FRANK L. KER, M.B., Ch.B. Fourth Edition. Pp. xvi., 354. London: Oxford University Press (Humphrey Milford). 1939. Price 12s. 6d.—This fourth edition of the original manual is revised by Dr. F. L. Ker, son of the original compiler. The revision has been done with varying success under the different sections. For example, the portion dealing with the present advances in the prophylaxis of measles and preventive measures in diphtheria is well and clearly expressed. On the other hand, the section dealing with the chemotherapy of erysipelas is so vague that the student would be little the wiser after reading it as to the drug or dosage to employ for this disease. The clinical section on diphtheria is almost unaltered since the First Edition in 1911. This is unfortunate, as since that time malignant diphtheria has occurred in many parts of Britain and Europe due to the gravis and intermediate strains of bacillus. Cases due to these strains have caused great difficulty in diagnosis to the practitioner owing to the slow appearance of the characteristic membrane. No reference whatever is made to this type of disease—a serious omission—nor would most authorities agree with 24,000 units as the maximum dose of antitoxin. For the "acidosis of diphtheria" alkalies are recommended instead of intravenous glucose, which is coming into general use. Apart from these criticisms, the subject matter is well set out. The addition of a table of infectious diseases at the end is useful for students preparing for examinations, and one can recommend the manual to those commencing their studies of these diseases. The indexing is well done, but there is a complete absence of any references for those wishing to delve deeper into the subjects discussed.

**Silicosis and Asbestosis.** By A. J. LANZA, M.D. Pp. xxvi., 439. New York: Oxford University Press. 1938. Price 25s.—This book is the work of six experts who were among the pioneers in the study and investigation of Silicosis and Asbestosis. The contributors, four of whom are American and two British, have approached the subject from a different angle, and the seven sections of the book deal with the History, Ætiology, Symptoms and Diagnosis, Roëntgen Ray Diagnosis, Pathology, Experimental Pathology, Occupational, Preventive and Legislative Aspects in Great Britain, and Public Health and Economic Aspects in the United States of America. The result is probably the most authoritative, complete and up-to-date treatise on this most important industrial class of disease. In spite of the special nature of the subject, all the sections are very readable and the type and illustrations are excellent. An extensive bibliography is appended at the end of each section and there is a good index. It is inevitable that a certain amount of repetition should occur in the different sections, but this does not detract from their value. The only criticism one has to make is the presence of rather more printers' errors than one expects to find. These will no doubt be corrected in a future edition.

**Personality Development and Social Control in Terms of Constitution and Culture.** By IRA S. WILE, M.S., M.D. Pp. x, 57. London: Oxford University Press (Humphrey Milford). 1939. Price 3s. 6d.—One of the characteristics of post-war medicine is the conception that many "Diseases" can only be understood in relation to their social and economic settings, and that the patient's personality plays a vital part in this relationship. Sir Humphrey Rolleston and other writers have termed this movement "Neo-Hippocratic," and actually there is a revival of the philosophic approach to illness that characterized Greek medicine. This movement is associated with a considerable difference of opinion in details between the various schools, which is in reality evidence of a healthy spirit of criticism. Dr. Wile has reviewed this philosophic development in the course of four lectures that were delivered in London in 1937, which show all the hall-marks of deep thinking on current problems. He is particularly interesting on personality in relation to culture, and the influence of urban civilization is well summarized in his words: "Man's continued growth away from the soil has been attended with significant effects upon his structure and his capacity for adjustment to the crowd pressures he has multiplied. Cities create artificial conditions that tax the human body less, and the human mind more. Despite the advance in creature comforts, urban life is a tremendous force in causing a lessening of nervous stability. It is too late to return to early forms or rural life, and it is therefore essential to evaluate urban difficulties in the interest of a reasonable mitigation of their securities." His remedies involve a consideration of current economic conditions and he predicts a far greater degree of social planning, essentially biological in its approach. Altogether this is a most stimulating little book that should be read by all interested in the sociological implications of our profession: they will find no trite remedies but much food for thought.

**Play Therapy in Childhood.** By C. H. Rogerson, M.D. Pp. viii., 64. London: Oxford University Press (Humphrey Milford). 1939. Price 3s. 6d.—This short book is based on a study of thirty-six children during a two-year period. After giving a brief historical survey the author describes his own technique and summarises the play of four children. He then discusses in a straightforward but superficial way the relationship of the therapist to the child, the fantasies expressed in the play and the reasons for improvement. The views put forward represent a mid-way position between that of merely passive observation and the psychoanalytical approach with its transference situation and direct interpretation to the child of the inner meaning of the play. The book is clearly written and provides an excellent introduction to a subject which should be of interest to anyone concerned with children's psychological problems.

**Clinical Examination of the Nervous System.** By C. H. Monrad-Krohn, M.D., F.R.C.P. Seventh Edition. Pp. xvii., 319. Illustrated. London: H. K. Lewis & Co. Ltd. 1938. Price 8s. 6d.—That this book has now reached its seventh edition is sufficient to indicate its value. Previous editions have been reviewed from time to time in this *Journal*. The continual advance of clinical neurology has again necessitated a complete edition of the book. Some of the chapters have been partly rewritten with a large number of additions and alterations, bringing the work into line with the advance of modern knowledge. There are many fresh illustrations, particularly in relation to ventrilography and encephalography, as these methods of investigation are becoming increasingly important. Other additions are a description of angiography, and also the method of examination of unconscious patients; while both positive and negative X-ray pictures are included.

In spite of these additions the work has been kept as concise as possible, and forms a thoroughly good and reliable guide for complete investigation of the nervous system.

**Pye's Surgical Handicraft.** By twenty-five authors: edited by HAMILTON BAILEY, F.R.C.S. Eleventh Edition. Pp. viii., 512. Illustrated. Bristol: John Wright & Sons Ltd. 1939. Price 21s.—This excellent book was first published fifty-four years ago and it still retains its popularity. It has been used with profit by generations of house surgeons, and it should be read by every student as soon as he has qualified, because it sets out to give just those few extra points of information which turn a good student into a good house-surgeon. The hints given on the post-operative treatment of thyroidectomies, the treatment of abdominal distension, circumcision and even on medico-legal reports, are invaluable. There is a very human note at the end on how a resident should collaborate with the nursing staff. Mr. Hamilton Bailey has done a service to this generation of house surgeons in placing his wide knowledge of practical hints at their disposal, and the publishers have maintained their usual high standard, both of paper, printing and illustrations. Altogether, both editor and publishers are to be congratulated on a very useful piece of work, and we heartily commend it to all would-be residents.

**Cranio-Cerebral Injuries.** By D. MUNRO, M.D., F.A.C.S. Pp. xxviii., 412. Illustrated. New York: Oxford University Press. 1938. Price 21s.—This book gives a full account of the management of cranio-cerebral injuries. It is based on the procedure adopted at the Neuro-Surgical Clinic at the Boston City Hospital. The enormous number of cases reviewed and the eminence of the author make the book peculiarly acceptable to all who have to deal with these accidents. The book is a complete survey, commencing with the first aid and casualty treatment, and concluding with after-care and home convalescence. No detail has been overlooked. The methods described are thoroughly sound and can be well recommended. A chapter on nursing methods has been included and is very helpful. Obstetricians and midwives will appreciate a long and well-argued chapter on cranio-cerebral injuries in the new-born. From this chapter, as indeed from the whole book, one receives an impression of enthusiasm and meticulous care which is exceedingly stimulating. One is interested to note in a number of places the mention of decline in morbidity and mortality after the proper segregation of these cranio-cerebral injuries to the neuro-surgical service, an essential requirement in every large city. An interesting feature is the relegation of all case-histories properly referenced in the text to a separate chapter at the end of the book, where one hundred and six pages are devoted to case histories illustrating almost every variety of head injury. Detailed criticisms of the book can be shortly summarized as follows: The photographs are satisfactory but the diagrams are very small and therefore tedious to follow. The print and format is good—the usual standard of Oxford Medical Publications. An unfortunate Americanism is permitted in that numerals are used in the text instead of the usual spelling. It is irritating to read " such a reflex may be activated in 4 ways of which pain is 1." ! Oddly enough, the spelling is in the main English in type. These minor points are, however, minute blemishes in a really trustworthy and informative book.

**A Handbook of Midwifery.** By Sir COMYNS BERKELEY, M.C., M.D., F.R.C.P., F.R.C.S., F.R.C.O.G. Tenth Edition. Pp. x., 633. Illustrated. London: Cassel & Co. Ltd. 1938. Price 8s.—This book is well known and deservedly popular from its previous editions, particularly amongst obstetric dressers, pupil-midwives, and midwives, for whom it is intended. In the tenth edition the subject matter has been brought up to date and in large measure rewritten, whilst everything superfluous has been eliminated. The descriptions of various obstetrical emergencies and manipulations are excellent, whilst the clear statements of treatment by the midwife and treatment by the doctor, given under separate headings, are extremely valuable. The handy size of the book together with the clarity of the type should greatly add to its popularity. The illustrations are uniformly good and the whole format of the book most pleasing. Its index is thoroughly adequate, and the section at the end of the book on " a few points of law " shows the care that has been exercised to make it complete. It would be very hard to improve this as a practical handbook for reference and study.

**Dysmenorrhœa.** By A. A. Davis, M.D., Ch.M. Pp. xii., 254. Illustrated. London: Oxford University Press (Humphrey Milford). 1938. Price 12s. 6d.—The extensive employment of girls and women in industry makes dysmenorrhœa, though a minor disorder, of considerable economic importance to the nation, as well as to the individual, and the appearance of this monograph by an author who has done a considerable amount of research work into its ætiology and treatment is very welcome. Any advance in our knowledge of the causation and treatment of dysmenorrhœa must be of advantage to the medical profession, to the employer, and to the patient. The opening chapter of the book gives a short historical survey of the contributions made through the centuries to the medical knowledge of this disorder, and it is interesting to read that Hippocrates described, and used, dilatation of the cervix for the relief of menstrual pain, and that Soranus in 200 B.C. recommended rest in bed, hot drinks and baths, with exercises and massage in the intermenstrual period. The section of the book which deals with the association of dysmenorrhœa and hormonal disorders is concise, and the subject matter in line with modern advances in endocrinology. Several of the older theories which have been advanced to explain spasmodic dysmenorrhœa, *e.g.* hypoplasia of the uterus, and acute flexion of the uterus, are rejected by the author. Chapter six, which occupies nearly one-third of the book, is devoted to the neurogenic theory of spasmodic dysmenorrhœa ; it records the author's own investigations into the pathological changes in the autonomic nervous system associated with this type of dysmenorrhœa, as well as those of other workers in this field. This chapter is particularly interesting and is well illustrated. In the chapters dealing with treatment nothing very new is suggested, but the author is convinced of the value of presacral sympathectomy for the relief of pain in severe cases of spasmodic dysmenorrhœa. The book is furnished with a very complete bibliography, and can be recommended to practitioners wishing to have an up-to-date book on the subject.

**The Evolution of Obstetric Analgesia.** By A. M. CLAYE, M.D., F.R.C.S., F.R.C.O.G. Pp. x., 103. Illustrated. London : Oxford University Press (Humphrey Milford). 1939. Price 6s. —As an historical survey this book will be greatly welcomed. It is easily read and one passes eagerly from page to page and chapter to chapter. It is particularly interesting to read verbatim reports of Simpson's writings and lectures, and also of one humorously-written report of experiments with the newly-discovered chloroform. This book, however, is much more than a history of analgesics, for it describes in masterly fashion the advantages and disadvantages of the various analgesics, such as scopolamine, nitrous oxide, the barbiturates, etc., used since Simpson first tried ether in January, 1847, for a difficult obstetric case. Simpson's conclusions at that time could not be improved on even to-day: "It is obvious . . . he had in mind all the criteria for excellence which are still used." The author gives much clinical information, illustrated by tables largely from his own experience. The book is well indexed and references are appended to each chapter.

### Obituary

